# Comparative efficacy of exercise modes on cardiometabolic health in women with polycystic ovary syndrome: a systematic review with pairwise and network meta-analyses

**DOI:** 10.1186/s12905-025-04240-x

**Published:** 2026-01-03

**Authors:** Mousa Khalafi, Saeid Fatolahi, Ghodsieh Rahmatpanah, Michael E. Symonds, Sara K. Rosenkranz, Farnaz Dinizadeh, Alexios Batrakoulis

**Affiliations:** 1 Department of Sport Sciences, Faculty of Humanities, University of Kashan, Kashan, Iran; 2 Department of Physical Education and Sport Sciences, Faculty of Humanities, Tarbiat Modares University, Tehran, Iran; 3 Centre for Perinatal Research, Academic Unit of Population and Lifespan Sciences, School of Medicine, University of Nottingham, Nottingham, UK; 4 Department of Kinesiology and Nutrition Sciences, University of Nevada Las Vegas, Las Vegas, NV USA; 5 Department of Sport Sciences, Tabriz branch, Azad University, Tabriz, Iran; 6 Department of Physical Education and Sport Science, Democritus University of Thrace, Komotini, Greece; 7 Department of Physical Education and Sport Science, University of Thessaly, Trikala, Greece

**Keywords:** Exercise training, Insulin resistance, Lipid profile, Inflammation, Polycystic ovary syndrome

## Abstract

**Introduction and aim:**

Although exercise training is beneficial to patients with polycystic ovary syndrome (PCOS), its impact on cardiometabolic health remains uncertain. Therefore, the objective of the current systematic review with pairwise and network meta-analyses was to investigate the efficacy of different modes of exercise in improving glucose homeostasis, lipid profiles, and systemic inflammation.

**Methods:**

Three electronic databases, including PubMed, Web of Science, and Scopus were searched in the English language for publications from inception to July 2025. Randomized control trials comparing the effects of exercise training, including aerobic (AT), resistance (RT), combined (CT), or high-intensity interval training (HIIT), Yoga/Tai Chi with non-exercise controls (CON) or another exercise modality on markers of glucose homeostasis, lipid profiles, and C-reactive protein (CRP) were included in the meta-analyses. Weighted mean differences (WMD) or standardized mean differences (SMD) along with 95% confidence intervals (95% CIs), were determined using random-effects models.

**Results:**

Twenty-five studies involving 1, 107 participants with mean ages from 18 to 34 years, were included. Exercise training reduced fasting insulin [SMD: -0.28, *p* = 0.01], Homeostatic Model Assessment of Insulin Resistance (HOMA-IR) [SMD: -0.50, *p* = 0.008], triglycerides (TG) [WMD: -2.70 mg/dl, *p* = 0.01], total cholesterol (TC) [WMD: -3.74 mg/dl, *p* = 0.04], and CRP [SMD: -0.61, *p* = 0.001], significantly more than CON, but no significant differences were observed for fasting glucose, low-density lipoprotein (LDL), or high-density lipoprotein (HDL). Based on network meta-analysis, Yoga/Tai Chi reduced fasting glucose [WMD: -5.54 mg/dL, *p* = 0.001], AT reduced HOMA-IR [SMD: -0.57, *p* = 0.03], and CRP [SMD: -0.82, *p* = 0.001], HIIT reduced fasting insulin [SMD: -0.38, *p* = 0.03], HOMA-IR [SMD: -0.56, *p* = 0.05] and LDL [WMD: -6.45 mg/dl, *p* = 0.01], and CT reduced TG [WMD: -3.61 mg/dl, *p* = 0.004] and LDL [WMD: -8.40 mg/dl, *p* = 0.01] significantly more than CON.

**Conclusion:**

Exercise training may be effective for improving glycemic control, lipid profiles, and systemic inflammation among women with PCOS. Among exercise modes examined, AT and HIIT appeared to be the most effective for improving glucose homeostasis, AT for reducing systemic inflammation, and CT for improving lipid profiles (PROSPERO ID: CRD420251127315).

**Supplementary Information:**

The online version contains supplementary material available at 10.1186/s12905-025-04240-x.

## Introduction

 Polycystic ovary syndrome (PCOS) is the most prevalent endocrine condition among women of reproductive age [1]. The prevalence of PCOS varies depending on the diagnostic criteria applied, with estimates ranging from 6 to 10%. Typically, a diagnosis of PCOS is determined by the presence of a combination of clinical signs of menstrual irregularities or anovulation, clinical or biochemical hyperandrogenism, and polycystic ovaries [2]. PCOS is a distinct female reproductive risk factor for cardiometabolic diseases, including obesity, type 2 diabetes, dyslipidemia, hypertension, metabolic syndrome, myocardial infarction, and stroke; the primary causes of female mortality [3]. Obesity, a significant modifiable risk factor for cardiometabolic diseases, is present in ~ 50% of PCOS cases [4], but whether PCOS is caused by obesity remains to be established [5], as is its independent contribution to cardiometabolic diseases [6]. Women diagnosed with PCOS, however, exhibit insulin resistance, irrespective of their body mass index (BMI), suggesting a heightened risk of metabolic dysregulation, even in the absence of obesity [7].

A substantial body of evidence demonstrates a robust correlation between PCOS and cardiometabolic health impairments [8–12], with lifestyle interventions, including exercise training, likely to be beneficial for women with PCOS. In this regard, exercise training is regarded as a primary lifestyle intervention for PCOS management, inducing therapeutic effects on various cardiometabolic health outcomes [13]. International guidelines recommend that women with PCOS perform 150–300 min of moderate-intensity or 75–150 min of vigorous-intensity aerobic training (AT) weekly, or a similar combination, spread evenly across the week, plus resistance training (RT) on two non-consecutive days to remain healthy and prevent weight gain [13, 14]. Training that combines aerobic and resistance training (CT) also improves cardiometabolic health in women with PCOS [13, 15–18]. Additionally, low-volume high-intensity interval training (HIIT), when used as a standalone intervention, reduces insulin resistance and BMI in women with PCOS, but it has not been established whether it impacts other cardiometabolic health outcomes as compared with other modes of exercise or with a control [19, 20].

Despite the plethora of systematic reviews and pairwise meta-analyses demonstrating the significance of exercise in enhancing cardiovascular and metabolic health parameters [13, 15–18, 21, 22], its efficacy on cardiometabolic health outcomes, such as glucose or lipid metabolism, and chronic inflammation, remains uncertain. Consequently, the present network meta-analysis employs a methodological framework that extends pairwise meta-analysis by allowing for the comparison and ranking of multiple interventions using both direct and indirect evidence, even in the absence of head-to-head trials. This approach aims to address the identified knowledge gap by evaluating and comparing the efficacy of different modes of exercise in improving metabolic homeostasis and systemic inflammation in women with PCOS.

## Methods

This systematic review with pairwise and network meta-analyses, was prospectively registered in the International Prospective Register of Systematic Reviews (PROSPERO ID: CRD420251127315). It was performed based on the Preferred Reporting Items for Systematic Reviews and Meta-analyses (PRISMA) guidelines and the Cochrane Handbook for Systematic Reviews of Interventions.

### Search strategy

Systematic literature searches in three primary electronic databases, including PubMed, Web of Science, and Scopus were conducted between inception through July 2025. The search strategy used the Boolean operators “AND” and “OR” between the following key words: (“Polycystic Ovary Syndrome” or “Ovary Syndrome” or “Ovarian Syndrome” or “Stein Leventhal syndrome” or “polycystic ovary disease” or “pcos”) and (“exercise” OR “training” OR “exercise training” OR “physical activity”). In addition, the reference lists of eligible studies, plus Google Scholar and previously published meta-analyses [23] were manually searched for additional studies that may have been missed. The searches were conducted by one author (M Kh).

### Eligibility criteria and study selection

To be included in the review, studies must have been published in the English language, in peer-reviewed journals, according to the PICOS (Population, Intervention, Comparison, Outcome, and Study design) framework. For the population, studies involving women with mean ages ≥ 18 years who were diagnosed with PCOS based on the Rotterdam criteria or the National Institutes of Health (NIH) criteria were included. For the intervention, any mode of exercise training, including aerobic training, resistance training, combined (aerobic and resistance) training, high-intensity interval training, and Yoga/Tai Chi with intervention durations ≥ 2 weeks and weekly sessions ≥ 2, were included. Studies were required to include exercise interventions lasting at least two weeks, since meaningful cardiometabolic adaptations in women with PCOS typically need to be this length, consistent with previous PCOS-specific reviews [24]. For the comparison, studies with non-exercise control groups (CON) or comparisons with other exercise modalities were included. For the outcomes, markers of glucose homeostasis, including fasting glucose, fasting insulin, and insulin resistance measured using the HOMA-IR method; lipid profiles including triglycerides (TG), total cholesterol (TC), low-density lipoprotein cholesterol (LDL), and high-density lipoprotein cholesterol (HDL); and the commonly used marker of systemic inflammation, C-reactive protein (CRP), were included. For the study design, only randomized controlled trials (RCTs) or randomized clinical trials were eligible for inclusion. Excluded studies included non-original studies, studies involving non-Human animals, publications that duplicated studies, non-randomized controlled trials, observational studies. In addition, according to the Cochrane Handbook, heterogeneity in intervention type can bias pooled results; therefore, exercise‑plus‑supplement interventions were excluded [25]. However, studies that combined exercise with dietary interventions, when compared with dietary interventions alone, were included because diet, like exercise, imposes a metabolic load and allows clearer interpretation of exercise effects [26, 27], whereas exercise-plus-supplement studies were excluded due to the heterogeneous mechanisms recruited. All retrieved studies were imported into EndNote (Version 21), and after removing duplicate records, screening was conducted in two stages. First, all studies were screened by titles and abstracts, and subsequently, the remaining full-text records were assessed against the apriori inclusion and exclusion criteria. The study selection process was conducted by two independent authors (G.R. and F.D.), and any disagreements were resolved through discussion with other authors (M.Kh. and A.B.).

### Data extraction

Two authors (S.F. and G.R.) independently extracted data from all included studies, with any conflicts resolved by discussion with a third author (M.Kh.). The following data were extracted: first author name and year of publication; participant characteristics, including ages, BMIs, and health statuses; intervention characteristics, including exercise modes, intensities, durations, and protocols; and outcomes of interest, including glycemic markers, lipid profiles, and CRP. To perform meta-analyses, mean changes (post-intervention values minus pre-intervention values) and their related standard deviations (SDs), along with sample sizes, were extracted. When required, these data were calculated from pre- and post-intervention values using the appropriate formula [28]. Additionally, data were calculated from other reported statistical presentations, such as medians and interquartile ranges (IQRs), standard errors, or confidence intervals, when required using established and validated methods [29–31]. GetData software was also used to extract data from figures when necessary. If studies had multiple exercise interventions using different modes, the different interventions were included as separate arms, and sample sizes of the control groups were divided by the number of comparison interventions. If insufficient data were reported, the corresponding authors were contacted via email for studies published within the last 5 years.

### Quality assessment

Two independent authors (G.R. and F.D.) assessed the methodological quality of included studies using the Physiotherapy Evidence Database (PEDro) scale. Any discrepancies between the authors were resolved through discussion with a third author (M.Kh.). The PEDro scale is a validated tool for evaluating the methodological quality of randomized control trials, and consists of eleven items. However, two items, including blinding of participants and therapists, were excluded from the overall scores due to the impossibility of blinding participants and therapists for exercise interventions. The methodological quality of the included studies ranges from 0 to 8 (Supplementary Table 1).

### Meta-analyses

Pairwise meta-analysis was performed using Comprehensive Meta-Analysis Software (CMA, Biostat, Version 3). The pooled synthesis for the comparison between exercise training and CON was calculated using standardized mean differences (SMD) when outcomes were reported in different measurement units, or using weighted mean differences (WMD) when outcomes were reported in the same units. All effect sizes were reported with their respective 95% confidence intervals (95% CIs). We used random-effects models to pool the effect sizes and to generate the funnel and forest plots. Heterogeneity was assessed using Q statistics and I^2^ values, where I^2^ values ≤ 25%, 25 − 75%, and ≥ 75% were interpreted as low, moderate, and high heterogeneity, respectively. Publication bias was assessed using the visual interpretation of funnel plots and Egger’s tests, with p-values < 0.05 considered as statistically significant. To clarify the effects of exercise mode on each outcome, network meta-analysis was performed in R Statistical Software (v 4.5.1; R core team 2025) using the netmeta package. Similar to pairwise meta-analysis, SMDs or WMDs with corresponding 95% CIs were calculated using both direct, indirect and pooled comparisons, through a random effects model within a frequentist framework. To explore comparative relationships between intervention arms, network geometry plots were generated. Forest plots and league tables were created to pool and present the effect sizes. Interventions were ranked using the P-score ranking, which ranged from 0 to 1, with the higher scores representing the exercise intervention that was likely to be the most effective for the outcomes of interest. To assess heterogeneity, tau scores, Q statistics, and I^2^ values were calculated, with I^2^ values ≤ 25%, 25 − 75%, and ≥ 75% interpreted as low, moderate, and high heterogeneity, respectively. To assess the network consistency assumption, global consistency was evaluated using a full design-by-treatment interaction random-effects model, while local consistency was assessed using the node-splitting method, which compares direct and indirect evidence. To assess publication bias, Egger’s tests was used with p-values < 0.05 considered statistically significant.

## Results

### Study characteristics

**Fig. 1:**
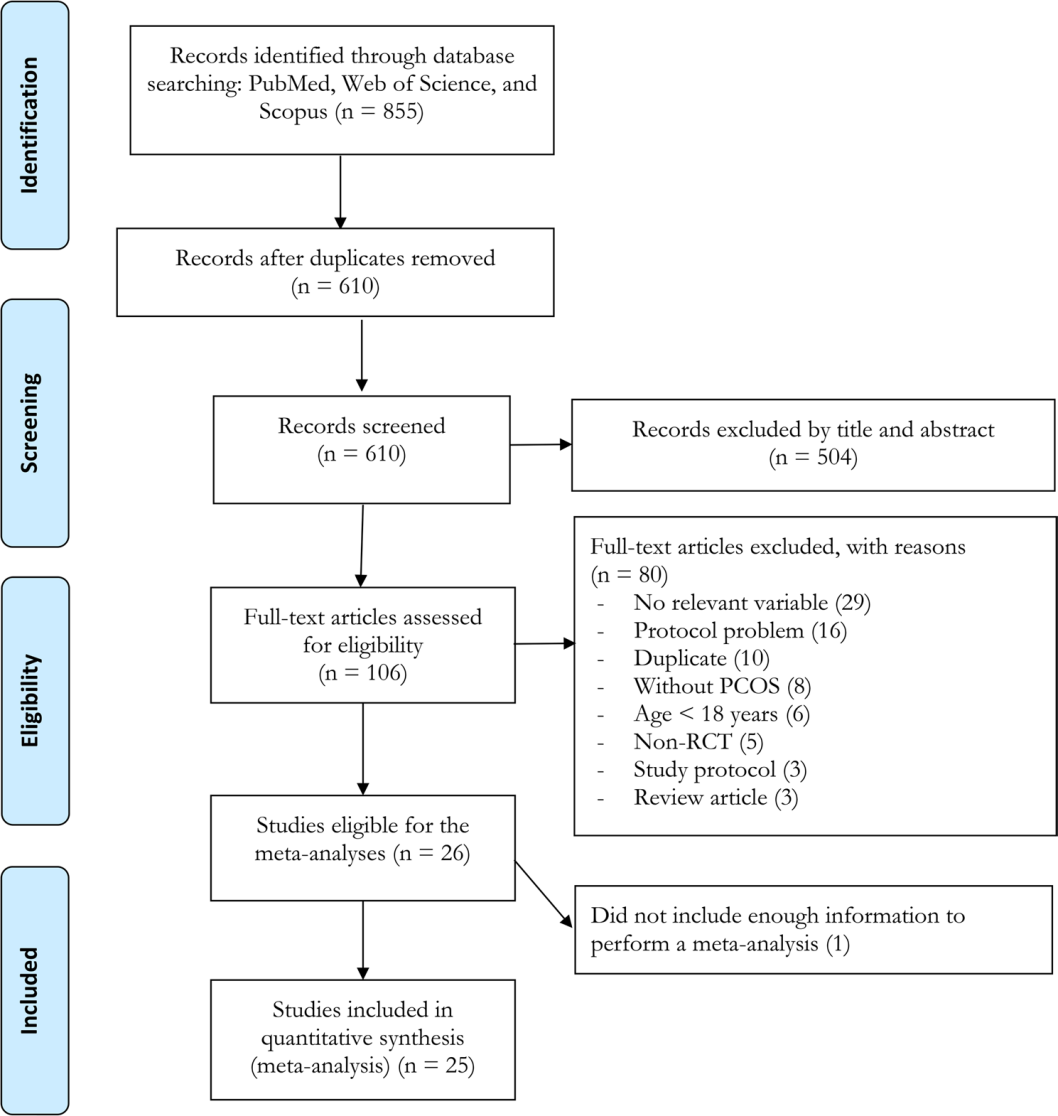
Flow diagram of systematic literature search

A total of 855 records were identified from the initial database searches, of which 610 articles remained after removing duplicate records. After reviewing the titles and abstracts, 504 articles were excluded. Subsequently, 80 additional articles were excluded after full-text review, and 1 study was excluded because it did not provide sufficient data. Ultimately, 25 randomized trials met al.l inclusion criteria, and were included in the meta-analyses (Fig. 1) [32–56]. A total of 1,107 women who had PCOS with mean ages ranging from 18 to 34 years, and mean BMIs ranging from 22 to 42 kg.m^2^, were included. A variety of exercise modalities were utilized as interventions, encompassing AT, RT, CT, HIIT, and Yoga/Tai Chi. The duration of the included interventions ranged from 8 to 24 weeks (Table 1).

**Table 1:** Characteristics of participants and interventions.

First author, year	Sample size	Participants characteristics	Age (years)	BMI (kg/m^2^)	Exercise training characteristics				Inflammatory markers and adipokines
					Protocol	Mode	Exercise vs. comparison group	Durations (Freq d/wk)	
Almenning et al. 2015 [45]	25	PCOS	27.2 ± 5.5	RT: 27.4 ± 6.9 HIIT: 26.1 ± 6.5 CON: 26.5 ± 5	HIIT: treadmill or walking or running and or cycling, 4 × 4 min at 90–95% of HR_max_, between intervals rest: 3 min at 70% of HR_max_ + 1d/wk: 10 × 1 min at all-out effort, between intervals rest: 1 min of passive or very low activity. RT: eight dynamic strength drills, 3 × 10 reps at 75% of 1-RM, between sets rest: 1 min	RT HIIT	RT, HIIT, CON	10-wk (2) Supervised	BMI, FBG, Insulin, HOMA-IR, TG, TCH, HDL, LDL, CRP
Benham et al. 2021 [46]	47	PCOS	HIIT: 29.1 ± 4.1 AT: 29.5 ± 4.6 CON: 29.1 ± 5.4	HIIT: 31.4 ± 8.6 AT: 31.3 ± 9.0 CON: 31.6 ± 8.2	HIIT: Aerobic exercise equipment, 10 × 30 s at 90% of HRR + 90 s of low intensity of AT. AT: 40 min at 50–60% of HRR	HIIT AT	HIIT, AT, CON	24-wk (3)	BMI, FBG, Insulin, TCH, TG, HDL, LDL
Bruner et al. 2006 [44]	12	PCOS	COM: 32.3 ± 2.64 CON: 28.4 ± 6.03	COM: 36.2 ± 5.29 CON: 37.1 ± 7.6	COM: AT: treadmill or cycling, 30 min at 70–80% of HR_max_, + RT: wight machines or free weight, 12 different exercises for major muscle groups	COM	COM, CON	12-wk (3)	BMI, Insulin
Costa et al. 2018 [33]	27	PCOS/Overweight	AT: 27.6 ± 4.5 CON: 24.4 ± 5	AT: 32 ± 4.2 CON: 33.6 ± 5.1	walking and/or jogging, 40 min, 60–85% of HR_max_	AT	AT, CON	16-wk (3) Supervised	BMI, FBG, Insulin, HOMA-IR, TG, TCH, LDL, HDL, CRP
Elbandrawy et al. 2022 [41]	40	PCOS	AT: 29.4 ± 3.45 CON: 30.66 ± 2.96	AT: 28.55 ± 1.36 CON: 28.62 ± 2.20	60 min, 60–70% of HR_max_	AT	AT CON	12-wk (3) Supervised	CRP
Kiel et al. 2022 [48]	64	PCOS	LV-HIIT: 30.4 ± 5.0 HV-HIIT: 30.1 ± 4.9 CON: 28.3 ± 5.3	LV-HIIT: 29.5 ± 5.7 HV-HIIT: 30.8 ± 7.2 CON: 31.2 ± 6.7	LV-HIIT: 10 × 1 min of maximal intensity, between sets rest: 1 min of low-intensity walking or passive recovery. HV-HIIT: 4 × 4 min at 90–95% of HR_max,_ between sets rest: 3 min at low-to-moderate intensity.	HIIT	LV-HIIT HV-HIIT CON	16-wk (3) Mix (Semi-Supervision)	BMI, FBG, Insulin, HOMA-IR, TG, TCH, LDL, HDL
Kirthika et al. 2019 [40]	30	PCOS	18–25	NG	45 min, brisk jogging in a treadmill, speed: 6 km/hour speed and. CON: Active stretching: upper and lower body, 20 s, 10reps.	AT	AT CON	12-wk (3)	HOMA-IR, CRP
Konopka et al., 2015 [38]	25	PCOS/Obese	33 ± 5	35 ± 5	60 min, cycling, 65% of VO_2peak_	AT	AT CON	12-wk (5) Supervised	BMI, FBG, Insulin, HOMA-IR
Li et al. 2022 [55]	42	PCOS	Tai Chi: 23.2 ± 4.38 AT: 22.9 ± 4.64	Tai Chi: 28.41 ± 4.03 AT: 29.57 ± 4.47	Tai Chi: 60 min, 24 forms of modified Yang style. AT (self-monitored): 60 min, brisk walking, cycling, jogging, or any other aerobic exercise	Tai Chi, AT (self-monitored)	Tai Chi, AT (self-monitored)	12-wk (3)	FBG, Insulin, HOMA-IR, TG, TCH, HDL, LDL
Lionett et al. 2021 [32]	64	PCOS	30 ± 5	30.5 ± 6.5	HV-HIIT: 4 × 4 min at 90–95% of HR_max_, between intervals rest: 3 min at 70% of HR_max_ + LV-HIIT: 10 × 1 min at maximal intensity, between intervals rest: 1 min at low intensity	HIIT	HV-HIIT LV-HIIT CON	16-wk (3)	FBG, Insulin, HOMA-IR
Lopes et al. 2018 [37]	110	PCOS	AT: 29.4 ± 4.1 HIIT: 30.2 ± 5.1 CON: 28.8 ± 6	AT: 29.3 ± 5.6 HIIT: 29 ± 4.8 CON: 29.9 ± 5.3	HIIT: 6–10 × 2 min, 70–90% of HR_max_, between intervals rest: 3 min, 60–70% of HR_max_. AT: 30–50 min, 65–80% of HR_max_	AT	HIIT AT CON	16-wk (3)	BMI
Mohammadi et al. 2023 [43]	28	PCOS	HIIT: 24.2 ± 4.8 CON: 22.9 ± 5.3	HIIT: 29.5 ± 4.5 CON: 31.4 ± 2.6	4–6 × 30 s, 100–110 maximum aerobic velocity, 4 laps, between laps rest: active 30 s, 50% of maximum aerobic speed	HIIT	HIIT CON	8-wk (3)	BMI, FBG, Insulin, HOMA-IR, TG, TCH, HDL, LDL, CRP
Nybacka et al. 2013 [53]	43	PCOS	Diet: 29.9 ± 5.5 COM + Diet: 31.8 ± 4.9	Diet: 35.4 ± 4.9 COM + Diet: 38.1 ± 7	45–60 min, walking, aerobics, jogging, swimming, and muscle strength training at a moderate to vigorous exertion level. Diet: Same in both groups, caloric intake: −600 kcal compared to pre-intervention. CHO: 55–60%, fat: 25–30% (with ≤ 10% saturated fat), protein: 10–15%	COM	COM + Diet Diet	16-wk (2–3) Supervised	BMI, FBG, Insulin, HOMA-IR
Orio et al. 2016 [51]	100	PCOS	AT: 25.9 ± 2.7 CON: 26 ± 2.6	AT: 26.7 ± 2.8 CON: 27 ± 2.9	45 min, bicycle ergometer, 60–70% of VO_2max_	AT	AT CON	24-wk (3) Supervised	FBG, Insulin, HOMA-IR, TCH, HDL, LDL, CRP
Patel et al. 2020 [52]	22	PCOS	Yoga: 30.9 ± 1.2 CON: 31.2 ± 2.3	Yoga: 35.1 ± 1.5 CON: 35.4 ± 3.3	Yoga, 60 min, pranayama exercises, vinyasa flow yoga, restorative yoga asanas, and meditation	Yoga	Yoga CON	12-wk (3)	BMI, FBG, Insulin, HOMA-IR
Patten et al. 2022 [49]	24	PCOS/Overweight	HIIT: 29.7 ± 4.8 AT: 32.5 ± 6.2	HIIT: 35.5 ± 6.8 AT: 38.4 ± 9.3	HIIT: two sessions: 12 × 1 min intervals at 90–100% of HR_peak_, between intervals rest: 1 min of recovery at a light load (active) + one session of 8 × 4 min intervals at 90–95% of HR_peak_, between intervals rest: 2 min light load (active). AT: 45 min, cycling, 60–75% of HR_peak_	HIIT AT	HIIT AT	12-wk (3) Supervised	BMI, FBG, Insulin, TG, TCH, LDL, HDL
Philbois et al. 2022 [36]	75	PCOS	HIIT: 29 ± 4 AT: 29 ± 5 CON: 29 ± 5	HIIT: 27.8 ± 4.2 AT: 27.7 ± 5.7 CON: 29.2 ± 5.4	AT: 50 min at 70–80% of HRres. HIIT: 35–45 min, 2 min intervals at 85–90%, between intervals rest: 3 min at 65–70% of HRres	HIIT AT	HIIT AT CON	16-wk (3) Supervised	BMI, FBG, Insulin, HOMA-IR, TG, TCH, LDL, HDL
Ribeiro et al. 2020 [42]	87	PCOS	AT: 29.1 ± 5.3 HIIT: 29.0 ± 4.3 CON: 28.5 ± 5.8	AT: 28.4 ± 5.6 HIIT: 28.7 ± 4.8 CON: 29.1 ± 5.2	AT: 30–50 min, 50–94% of HR_max_, HIIT: 30–50 min, 2 min intervals at 77–94% of HR_max_, between intervals rest: 3 min at 50–64% of HR_max_	AT HIIT	AT HIIT CON	16-wk (3)	BMI, FBG, Insulin, HOMA-IR, TG, TCH, HDL, LDL
Stener-Victorin et al. 2009 [50]	11	PCOS	AT: 30.4 ± 5.5 CON: 31.0 ± 3.2	AT: 26.8 ± 4.8 CON: 28.0 ± 6.2	30 min, Brisk walking, cycling or other aerobic exercise, faster than normal	AT	AT CON	16-wk (3)	BMI, FBG, Insulin, HOMA-IR, TG, TCH, LDL, HDL
Thomson et al. 2008 [39]	52	PCOS/Overweight/Obese	29.3 ± 6.8	36.1 ± 4.8	AT + Diet: walking, 25–45 min, 60–80% of HR_max_. COM + Diet: AT: walking, 3d/wk, 60–80% of HR_max_ + RT: 2d/wk, 5 exercise for major muscle groups, 3 sets, 12repsm 50–75% of 1-RM. CON (Diet): A 5000–6000 kJ/day energy-restricted high-protein meal plan (CHO:40%, fat: 30%, protein: 30%)	AT COM	AT COM CON (Diet)	20-wk (5)	FBG, Insulin, HOMA-IR, TG, TCH, HDL, LDL
Turan et al. 2015 [35]	30	PCOS	24.45 ± 2.8	COM: 21.8 ± 3.74 CON: 29.9 ± 4.4	COM: AT: Step (10–20 cm), 20 min, 60–70% of HR_max_ + RT: elastic bands, full-body exercises, 15reps, Borg RPE (5–6), between reps rest: 30s–1 min	COM	COM CON	8-wk (3)	BMI, FBG, Insulin, HOMA-IR, TG, TCH, HDL, LDL
Vigorito et al. 2007 [34]	90	PCOS/Overweight/	AT: 21.7 ± 2.3 CON: 21.9 ± 1.9	AT: 29.3 ± 2.9 CON: 29.4 ± 3.5	30 min, bicycle ergometer, 60–70% of VO_2max_	AT	AT CON	12-wk (3)	FBG, Insulin, TG, TCH, HDL, LDL, CRP
Vizza et al. 2016 [47]	15	PCOS	RT: 26 ± 7 CON: 29 ± 3	RT: 41.3 ± 12.5 CON: 34.0 ± 9.4	Supervised, 50 min, full-body, 2–3 sets, 8–12 reps + Homa-based: lower-intensity calisthenics exercises, 3 sets, 10 reps	RT	RT CON	12-wk (4)	FBG, Insulin, CRP
Wang et al. 2024 [56]	20	PCOS	HIIT: 32.4 ± 5.3 AT: 30.1 ± 6.3	HIIT: 33.7 ± 6.3 AT: 32.6 ± 7.5	HIIT: 15 min/d AT: 30 min/d, brisk walking	HIIT AT	HIIT AT	8-wk (5)	FBG, Insulin, HOMA-IR
Woodward et al. 2022 [54]	24	PCOS	AT: 29.7 ± 8.6 CON: 31.5 ± 5.5	AT: 35.8 ± 8 CON: 32.1 ± 7.3	60 min, cycle ergometer, elliptical trainer, rowing ergometer, or a motorized treadmill, 50–70% VO_2max_	AT	AT CON	12-wk (2–3)	FBG, Insulin, TCH, HDL

*Abbreviations*: *PCOS* Polycystic Ovary Syndrome, *HIIT* High-Intensity Interval Training, *AT* Aerobic Training, *RT* Resistance Training, *CON* Control, *COM* Combined Training (AT + RT), *LV-HIIT* Low-Volume High-Intensity Interval Training, *HV-HIIT* High-Volume High-Intensity Interval Training, *HR*_*max*_ Maximum Heart Rate, *HRR* Heart Rate Reserve, *HR*_*peak*_ Peak Heart Rate, *RM (1-RM)* One-Repetition Maximum, *VO*_*₂max*_ Maximal Oxygen Uptake, *VO₂peak* Peak Oxygen Uptake, *RPE (Borg RPE)* Rating of Perceived Exertion, *NG* Not Given/Not Specified, *BMI* Body Mass Index, *FBG* Fasting Blood Glucose, *HOMA-IR* Homeostatic Model Assessment of Insulin Resistance, *TCH* Total Cholesterol, *TG* Triglycerides, *LDL* Low-Density Lipoprotein, *HDL* High-Density Lipoprotein, *CRP* C-Reactive Protein, *CHO* Carbohydrate, *wk* Week, *d/wk* Days per week, *min* Minutes, *s (sec)* Seconds, *reps* Repetitions, *sets* Exercise Sets, *kcal* Kilocalorie

### Pairwise meta-analysis

#### Glucose homeostasis

Exercise training reduced fasting insulin [SMD: −0.28 (95% CI: −0.51 to −0.05), *p* = 0.01] and HOMA-IR [SMD: −0.50 (95% CI: −0.87 to −0.13), *p* = 0.008] significantly more than CON, but not fasting glucose [WMD: −0.52 mg/dL (95% CI: −1.91 to 0.86), *p* = 0.46] (Supplementary Figs. 1–3). There was significant heterogeneity amongst included studies for fasting glucose (I^2^ = 43.46, *p* = 0.001), fasting insulin (I^2^ = 57.60, *p* = 0.001), and HOMA-IR (I^2^ = 79.81, *p* = 0.001). Visual interpretation of funnel plots suggested publication bias, but the Egger’s test results did not confirm this bias for fasting glucose (*p* = 0.07), fasting insulin (*p* = 0.38), nor HOMA-IR (*p* = 0.12).

#### Lipid profiles

Exercise training reduced TG [WMD: −2.70 mg/dl (95% CI: −4.56 to −0.83), *p* = 0.005] and TC [WMD: −3.74 mg/dl (95% CI: −7.38 to −0.11), *p* = 0.04] significantly more than CON but changes were not significantly different for LDL [WMD: −0.22 mg/dl (95% CI: −7.21 to 6.76), *p* = 0.94] or HDL [WMD: 1.56 mg/dl (95% CI: −0.26 to 3.40), *p* = 0.09] (Supplementary Figs. 4–7). There was significant heterogeneity amongst included studies for LDL (I^2^ = 89.68, *p* = 0.001) TC (I^2^ = 42.81, *p* = 0.03), and HDL (I^2^ = 46.33, *p* = 0.02), but not for TG (I^2^ = 0.00, *p* = 0.91). Visual interpretation of funnel plots suggested publication bias, but the Egger’s test confirmed this bias for HDL only (*p* = 0.04), not for TG (*p* = 0.20), TC (*p* = 0.51), or LDL (*p* = 0.37).

#### Systemic inflammation

Exercise training reduced CRP [SMD: −0.61 (95% CI: −0.95 to −0.26), *p* = 0.001] significantly more than CON (Supplementary Fig. 8). There was significant heterogeneity amongst included studies for CRP (I^2^ = 52.15, *p* = 0.03). Visual interpretation of funnel plots suggested publication bias, but the Egger’s test did not confirm this bias (*p* = 0.72).

### Network meta-analysis

#### Glucose homeostasis

**Fig. 2:**
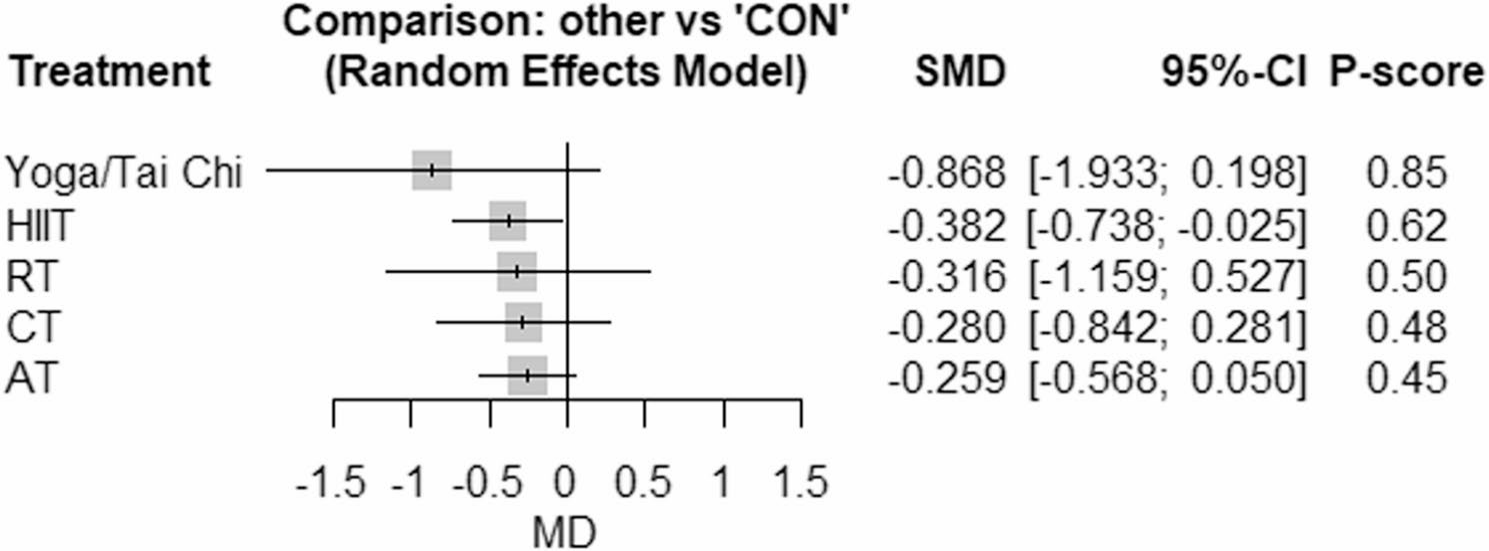
Forest plot of the network meta-analyses on fasting glucose. Data are reported as WMD (95% confidence limits). WMD: weighted mean difference

**Fig. 3:**
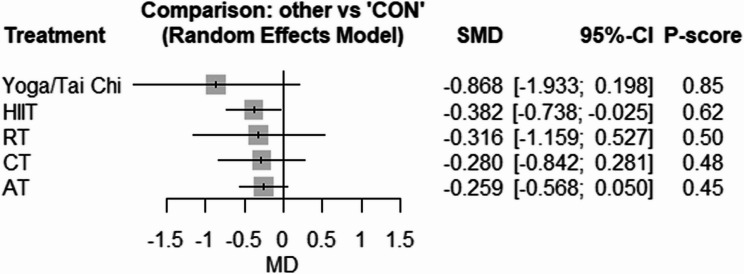
Forest plot of the network meta-analyses on fasting insulin. Data are reported as SMD (95% confidence limits). SMD: weighted mean difference

**Fig. 4:**
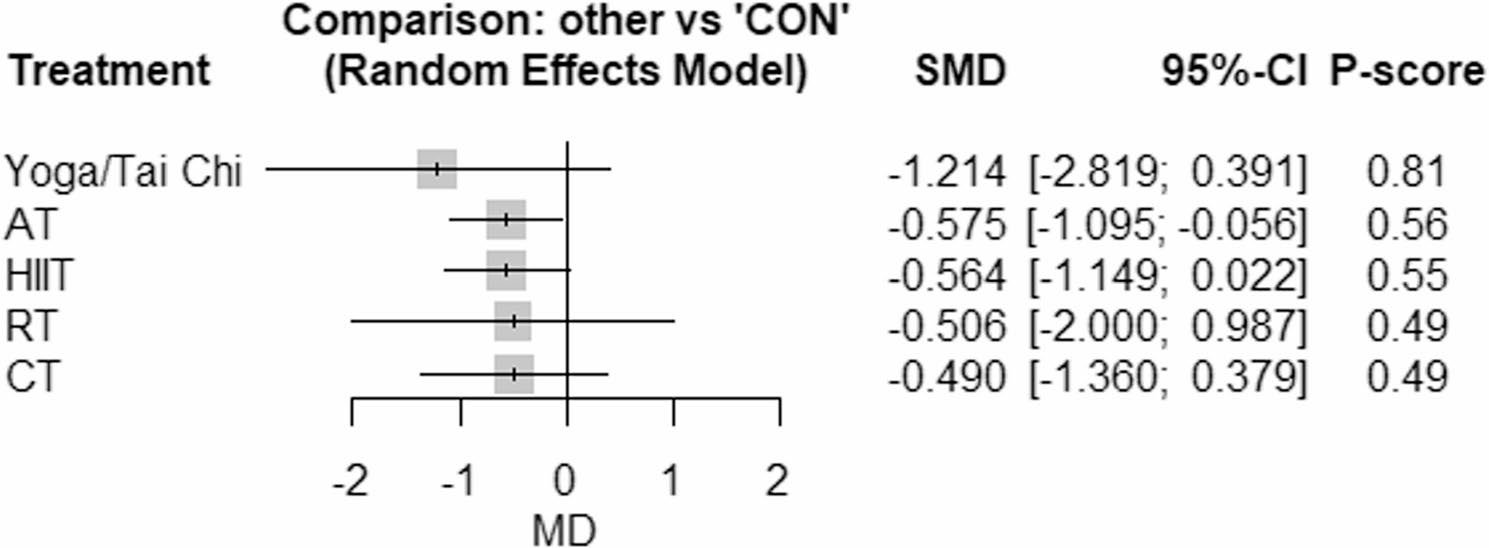
Forest plot of the network meta-analyses on insulin resistance. Data are reported as SMD (95% confidence limits). SMD: weighted mean difference

The network geometries for fasting glucose, fasting insulin, and HOMA-IR are provided in Supplementary Fig. 9–11. Compared with CON, only Yoga/Tai Chi [WMD: −5.54 mg/dL (95% CI −5.65 to −2.44), *p* = 0.001, P-score = 0.99] reduced fasting glucose significantly (Fig. 2). In the case of insulin, only HIIT [SMD: −0.38 (95% CI −0.73 to −0.02), *p* = 0.03, P-score = 0.62] effectively reduced fasting concentrations (Fig. 3). In addition, AT significantly reduced HOMA-IR [SMD: −0.57 (95% CI −1.09 to −0.05), *p* = 0.03, P-score = 0.56] and HIIT showed a near-significant reduction in HOMA-IR [SMD: −0.56 (95% CI −1.14 to 0.02), *p* = 0.05, P-score = 0.54] (Fig. 4). There was low to high heterogeneity amongst included studies for fasting insulin (tau^2 = 0.1690; tau = 0.4111; I^2 = 59.8% [35.9%; 74.8%]), HOMA-IR (tau^2 = 0.4981; tau = 0.7058; I^2 = 81.6% [71.2%; 88.3%]), and fasting glucose (tau^2 = 1.0702; tau = 1.0345; I^2 = 15.4% [0.0%; 49.2%]). Based on Q statistics to assess consistency under the assumption of a full design-by-treatment interaction random effects model, inconsistency was non-significant for fasting glucose (Q = 11.04, df = 8, *p* = 0.19), fasting insulin (Q = 2.94, df = 7, *p* = 0.89), and HOMA-IR (Q = 3.21, df = 6, *p* = 0.78). In addition, the results of the node splitting analysis, based on comparing direct and indirect evidence, showed no inconsistency for any outcome (supplementary Tables 2–4), except for glucose in the analysis of AT vs. CON. There was no significant publication bias based on the Egger’s test results for fasting insulin (*p* = 0.27), fasting glucose (*p* = 0.20), and HOMA-IR (*p* = 0.06) (Supplementary Figs. 12–14).

#### Lipid profiles

**Fig. 5:**
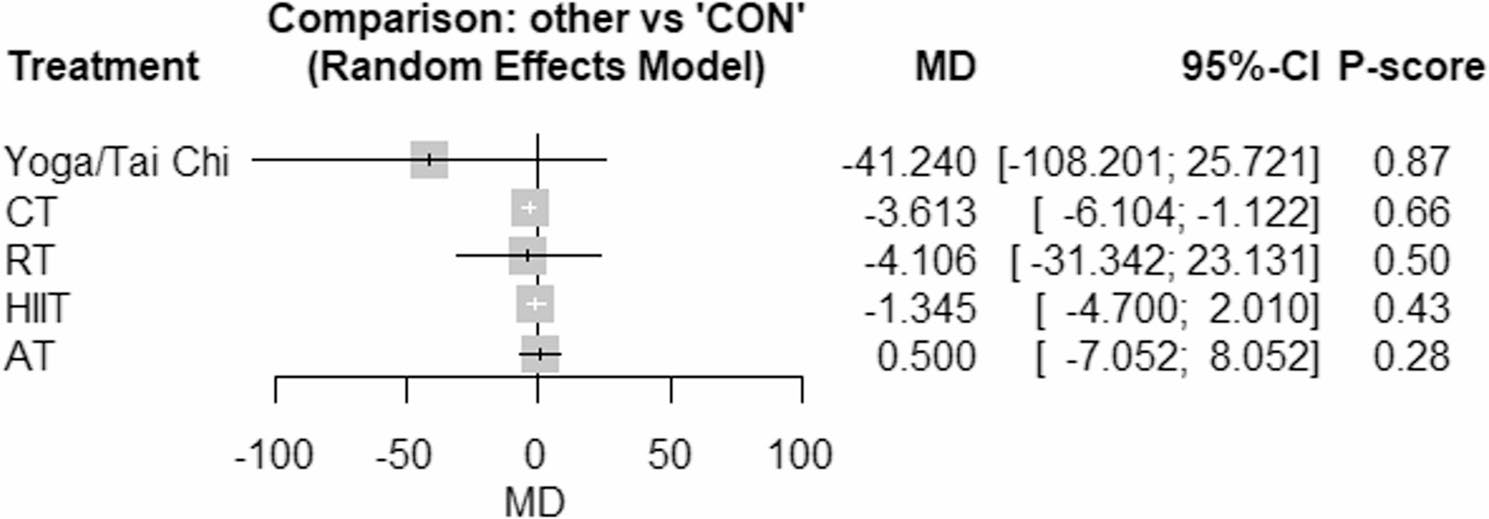
Forest plot of the network meta-analyses on TG. Data are reported as WMD (95% confidence limits). WMD: weighted mean difference

**Fig. 6:**
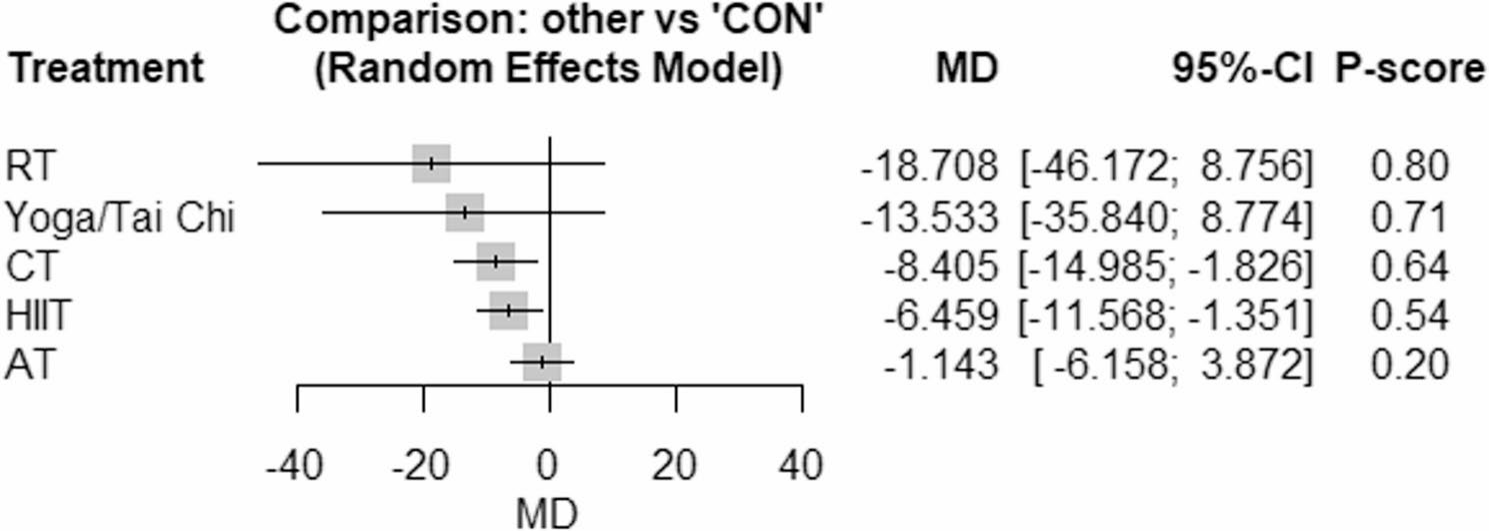
Forest plot of the network meta-analyses on LDL. Data are reported as WMD (95% confidence limits). WMD: weighted mean difference

**Fig. 7:**
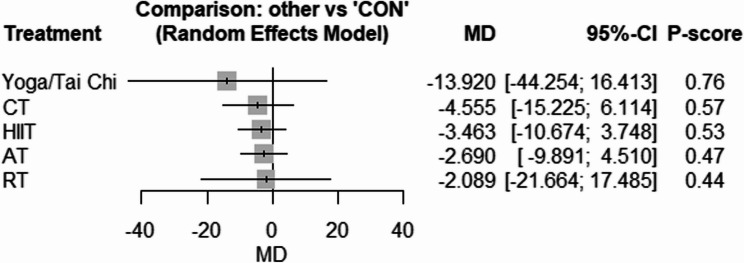
Forest plot of the network meta-analyses on TC. Data are reported as WMD (95% confidence limits). WMD: weighted mean difference

**Fig. 8:**
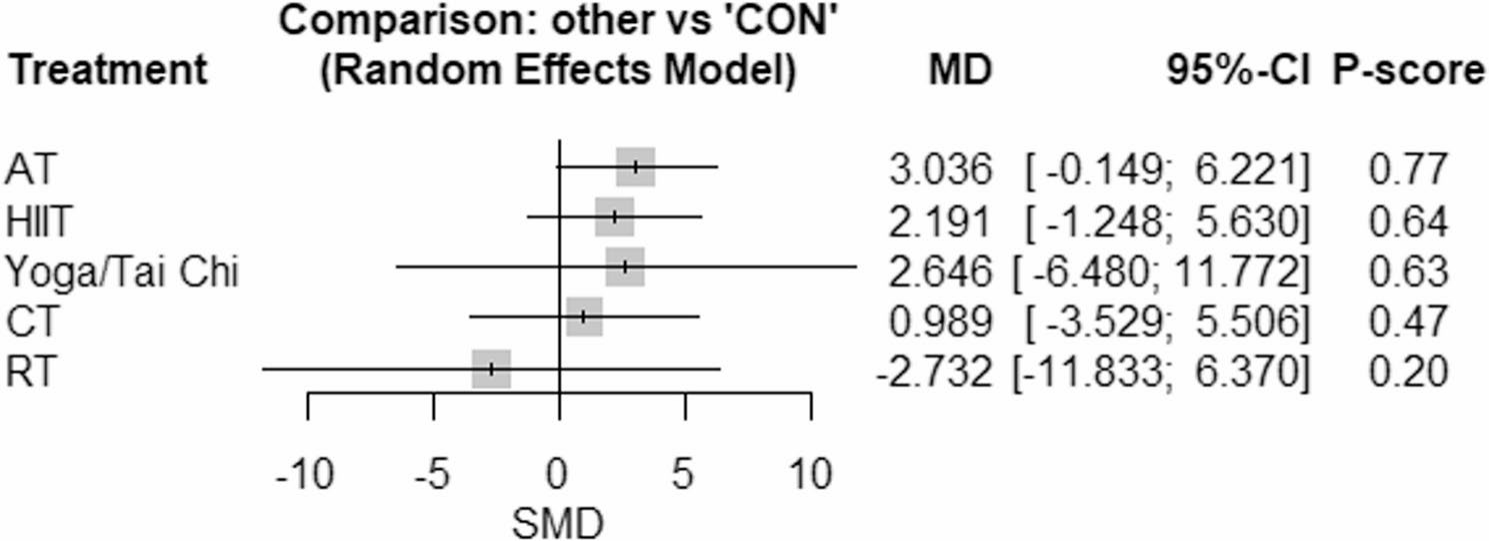
Forest plot of the network meta-analyses on HDL. Data are reported as WMD (95% confidence limits). WMD: weighted mean difference

The network geometries for fasting lipid profiles are provided in Supplementary Fig. 15–18. Compared with CON, only CT [WMD: −3.61 mg/dl (95% CI −6.10 to −1.12), *p* = 0.004, P-score = 0.66] effectively reduced TG (Fig. 5). In addition, CT [WMD: −8.40 mg/dl (95% CI −14.98 to −1.82), *p* = 0.01, P-score = 0.64] and HIIT [WMD: −6.45 mg/dl (95% CI −11.56 to −1.35), *p* = 0.01, P-score = 0.54] effectively reduced LDL (Fig. 6). However, no exercise intervention significantly changed TC or HDL as compared with CON (Figs. 7 and 8). There was low to high heterogeneity amongst included studies for TG (tau^2 = 0; tau = 0; I^2 = 0% [0.0%; 58.3%]), TC (tau^2 = 38.4045; tau = 6.1971; I^2 = 44.4% [0.0%; 70.3%]), LDL (tau^2 = 12.4750; tau = 3.5320; I^2 = 26.8% [0.0%; 61.3%]) and HDL (tau^2 = 9.0282; tau = 3.0047; I^2 = 52.2% [12.1%; 74.0%]). Based on Q statistics to assess consistency under the assumption of a full design-by-treatment interaction random effects model, inconsistency was non-significant for TG (Q = 1.62, df = 6, *p* = 0.95), TC (Q = 2.25, df = 6, *p* = 0.89), LDL (Q = 1.58, df = 6, *p* = 0.95), and HDL (Q = 2.67, df = 6, *p* = 0.84). Similarly, the results of the node splitting analysis, based on comparison of direct and indirect evidence, showed no inconsistency for all outcomes (Supplementary Tables 5–8). In addition, there was no significant publication bias based on the Egger’s tests for TG (*p* = 0.49), TC (*p* = 0.71), LDL (*p* = 0.06), nor HDL (*p* = 0.28) (Supplementary Figs. 19–22).

#### Systemic inflammation

**Fig. 9:**
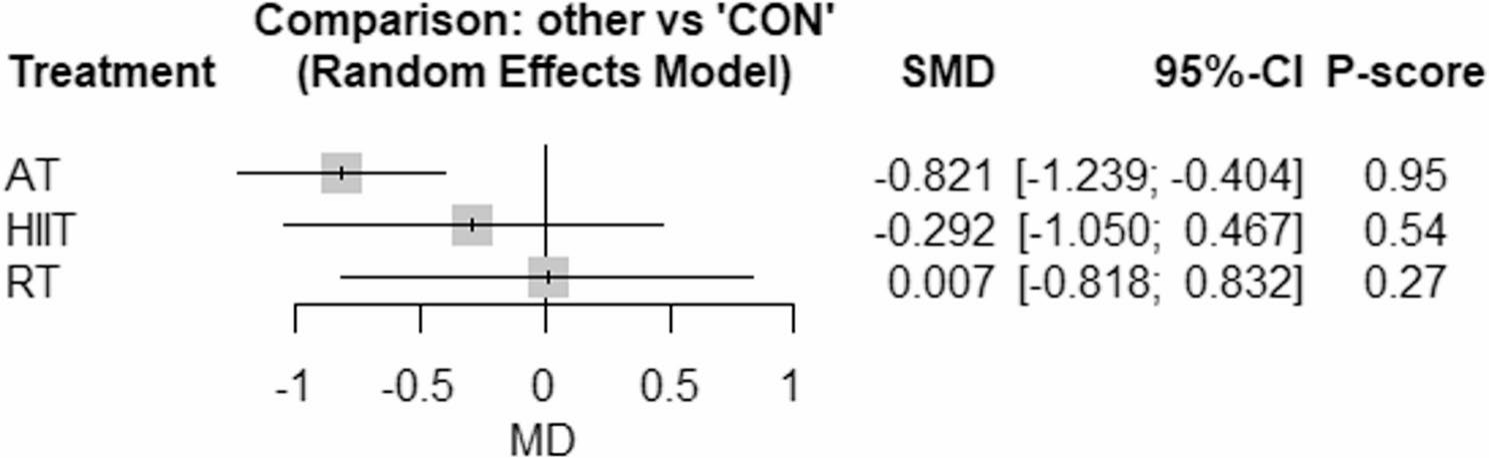
Forest plot of the network meta-analyses on CRP. Data are reported as SMD (95% confidence limits). SMD: weighted mean difference

The network geometry for CRP is provided in Supplementary Fig. 23. Compared with CON, only AT [SMD: −0.82 (95% CI −1.23 to −0.40), *p* = 0.001, P-score = 0.95] effectively reduced CRP (Fig. 9). There was low to high heterogeneity amongst included studies for CRP (tau^2 = 0.1307; tau = 0.3615; I^2 = 55.5% [0.0%; 80.9%]). Based on the Q statistic to assess consistency under the assumption of a full design-by-treatment interaction random effects model, there was no inconsistency for CRP (Q = 0.22, df = 2, *p* = 0.89). Similarly, the results of the node splitting analysis, based on comparing the direct and indirect evidence, showed no inconsistency for CRP (Supplementary Table 9). There was significant publication bias based on the Egger’s test for CRP (*p* = 0.04) (Supplementary Figs. 24).

## Discussion

### Summary of findings

This is the first systematic review and network meta-analysis of randomized and non-randomized trials to ascertain the effects of different modes of exercise on primary cardiometabolic health indicators in women with PCOS. We showed that HIIT, followed by AT, are likely to be the most effective for reducing insulin resistance, though neither improved fasting glucose. CT appeared to be the best exercise mode for lowering TG, while CT and then HIIT were the most effective mode for reducing LDL. AT was also the best for reducing CRP. Conversely, although RT had no obvious cardiometabolic benefits, our findings emphasize the potential of exercise training as a lifestyle intervention to prevent and/or treat cardiometabolic health complications associated with PCOS. Notably, while Yoga/Tai Chi demonstrated elevated P-scores in comparison to CON, these effects did not attain statistical significance. The Yoga/Tai Chi subgroup only comprised two studies, thereby limiting its statistical power and the establishment of definitive conclusions. Consequently, despite the elevated P-scores, it is premature to conclude that Yoga/Tai Chi represents the most efficacious exercise modality. The present review also highlights the importance of focusing on multiple modes of training for improving a range of cardiometabolic health markers. A mounting body of research has demonstrated that all the selected exercise modes improve glucose and lipid metabolism as well as chronic systemic inflammation through complementary skeletal muscle, adipose tissue, and hormonal adaptations [57].

### Glucose homeostasis

Impaired glucose control is a prevalent among women with PCOS [58], and increases the likelihood of metabolic dysregulation, thereby leading to the development of type 2 diabetes, metabolic syndrome, and cardiovascular diseases [1]. Regular exercise training is an effective lifestyle strategy for improving glucose homeostasis in this population [13], as it modulates insulin secretion, by improving pancreatic beta-cell function, and promotes insulin sensitivity [59]. Whilst ample evidence indicate that exercise enhances insulin sensitivity in individuals with diabetes and obesity [14, 60], conditions that are frequently observed along with PCOS [61], only a small number of studies show modest reductions in HOMA-IR among women with PCOS [39]. Our meta-analysis indicates that HIIT is likely to be the most effective exercise mode, followed by AT, for reducing fasting insulin and HOMA-IR in women with PCOS, results that are consistent in other clinical populations [17, 62–67]. In general, vigorous-intensity aerobic exercise has been shown to reduce the risk of metabolic syndrome more effectively than moderate-intensity aerobic activities [68]. However, the current meta-analysis did not demonstrate a beneficial effect of exercise on fasting glucose besides Yoga/Tai Chi, which appear to be effective meditative movement activities for improving this marker [69, 70]. Additionally, we were unable to study glycated hemoglobin. Overall, the current results indicate that exercise training, particularly HIIT and AT, may reduce insulin resistance [17], and this finding is consistent with previous meta-analyses investigating the effects of exercise on cardiometabolic health in similar populations [17, 19, 20]. The observed outcomes can be ascribed, at least in part, to the primary function of AT in enhancing insulin sensitivity and fat oxidation. This enhancement is achieved through the activation of AMP-activated protein kinase, the facilitation of glucose transporter protein translocation, the stimulation of mitochondrial biogenesis, and the reduction of visceral adiposity, consequently leading to a decrease in CRP [71].

### Lipid profiles

Lipid metabolism dysregulation is common in women with PCOS [72], increasing the risk of metabolic disturbances that may progress to cardiovascular disorders [1]. Exercise training is a promising strategy for improving blood lipid profiles in this population [73, 74], as it can impact enzymes and hormones which enable the mobilization of energy sources during extended periods of exercise. Changes in lipid metabolism with moderate exercise training have a positive effect on the cardiovascular system, but the precise mechanism remains to be fully elucidated [75]. Our network meta-analysis determined that CT is likely to be the most effective exercise modality for reducing TG in women with PCOS, while HIIT and CT were the top and second exercise modes, respectively, for lowering LDL. These outcomes are consistent with those reported in other populations with impaired cardiometabolic health [14, 60, 62, 63, 76–78]. Surprisingly, the exercise modalities we examined did not demonstrate significantly larger improvements in TC or HDL when compared to CON. Therefore, further high-quality trials are required to elucidate the absence of overall improvements in lipid profiles, and should concentrate on the mechanisms underlying the role of diverse exercise modalities in lipid profiles among women with PCOS [79]. Our findings, particularly those related to CT and HIIT, are consistent with previous meta-analyses on exercise and cardiometabolic health in women with PCOS [16, 17, 19, 20]. Moreover, the primary effects of RT include an increase in muscle mass and resting energy expenditure, as well as an expansion of glucose disposal capacity, that then contributes to modest improvements in lipid and inflammation status [60].

### Systemic inflammation

PCOS is frequently associated with elevated CRP, indicating the systemic low-grade inflammation that often occurs alongside excess visceral adiposity, endothelial dysfunction, hyperinsulinemia, and increased oxidative stress [80]. Current evidence demonstrates that exercise is an effective strategy for reducing CRP in women, particularly when body composition is improved [81]. In women with PCOS, exercise training improves CRP [15], although this finding is based on only a limited number of relevant trials. Consequently, further research is necessary to investigate the role of exercise in systemic inflammation among women with PCOS. It is noteworthy that AT appeared to be the most effective training modality, with a large effect size, according to our network analysis results. This finding underscores the pivotal role of AT in reducing inflammation when performed consistently, thereby supporting current international exercise prescription guidelines [13]. The primary mechanism through which AT reduces CRP involves the reduction of visceral fat, the enhancement of metabolic health, and the attenuation of inflammatory signaling. The efficacy of AT is most evident in individuals who initially present with elevated inflammation or suboptimal cardiometabolic health [82], as is the case with women diagnosed with PCOS [8]. Additionally, HIIT induces rapid metabolic benefits through enhanced AMPK activation and catecholamine responses, improving insulin sensitivity, reducing visceral and hepatic fat, and lowering CRP despite higher acute physiological stress [83, 84]. It has, however, been demonstrated that CT yields synergistic effects [62, 63], as a result of the simultaneous optimization of insulin sensitivity, muscle mass, and lipid metabolism [85]. Consequently, this approach consistently improves glycemic control and reduces CRP [86].

#### Limitations and future directions

The present study has several limitations. It is important to note that the available data did not examine the role of different exercise modes on additional key health parameters, such as cardiorespiratory fitness, blood pressure, or body composition. Furthermore, the presence of chronic low-grade inflammation was investigated only by evaluating CRP. It is also important to acknowledge the potential for residual heterogeneity, which may be attributable to variations in exercise protocols, participant characteristics, and study quality. Nevertheless, additional pertinent markers are required to more thoroughly assess this aspect of cardiometabolic health. These limitations leave a gap in our understanding of a wider range of cardiovascular disease risk factors and metabolic health markers in this population. further high-quality studies are needed to evaluate the long-term outcomes, safety, and effectiveness of exercise in women with PCOS, particularly regarding additional cardiometabolic health markers. In addition, the present data were based on trials lasting eight to twenty weeks. Therefore, long-term exercise interventions are warranted in future research to determine the longitudinal effects of diverse training modalities for improving cardiometabolic health. Finally, considerable heterogeneity and potential publication bias were observed for some outcomes, which should be considered when interpreting the results.

## Conclusion

The results of this systematic review with pairwise and network meta-analyses indicate that exercise training provides substantial benefits for selected cardiometabolic health indices in women with PCOS. The present findings underscore the significance of a multifaceted training approach aimed at enhancing a variety of cardiometabolic health markers, thereby assisting clinicians and practitioners in understanding how to support this population. Specifically, HIIT, followed by AT, are the modes that are most likely to be effective for reducing insulin resistance. CT is likely to be the most effective for lowering TG, while CT and HIIT ranked first and second for reducing LDL. Despite the good methodological quality of the trials included in our analysis, further high-quality research is necessary to assess long-term outcomes, safety, and effectiveness of exercise in women with PCOS. Further elucidation of the effective exercise modes, durations, and frequencies of exercise is needed to improve the integration of diverse exercise modalities into tailored multicomponent programs in this population.

## Supplementary Information

Supplementary Material 1.

## Data Availability

The datasets generated and/or analyzed during the current study are available from the corresponding author upon reasonable request.
