# Supplementary material for: Comparative efficacy of exercise modes on cardiometabolic health in women with polycystic ovary syndrome: a systematic review with pairwise and network meta-analyses
Source: BMC Womens Health. 2026 Jan 3;26:70. doi: 10.1186/s12905-025-04240-x (PMC12870144; doi:10.1186/s12905-025-04240-x)
Supplement: Supplementary file 1 — Supplementary Material 1. [file 12905_2025_4240_MOESM1_ESM.docx]

**Comparative efficacy of exercise modes on cardiometabolic health in women with polycystic ovary syndrome: a systematic review with pairwise and network meta-analyses**

**Running title:** **exercise training and polycystic ovary syndrome**

Mousa Khalafi^1*^, Saeid Fatolahi^2^, Ghodsieh Rahmatpanah^1^, Michael E Symonds^3^, Sara K Rosenkranz^4^, Farnaz Dinizadeh^5^, Alexios Batrakoulis^6,7*^

^1^Department of Sport Sciences, Faculty of Humanities, University of Kashan, Kashan, Iran

^2^Department of Physical Education and Sport Sciences, Faculty of Humanities, Tarbiat Modares University, Tehran, Iran

^3^Centre for Perinatal Research, Academic Unit of Population and Lifespan Sciences, School of Medicine, University of Nottingham, Nottingham, UK

^4^Department of Kinesiology and Nutrition Sciences, University of Nevada Las Vegas, Las Vegas, NV, USA

^5^Department of Sport Sciences, Tabriz branch, Azad University, Tabriz, Iran

^6^Department of Physical Education and Sport Science, Democritus University of Thrace, Komotini, Greece

^7^Department of Physical Education and Sport Science, University of Thessaly, Trikala, Greece

***Corresponding authors**

**Mousa Khalafi,** Department of Sport Sciences, Faculty of Humanities, University of Kashan, Kashan, Iran; Email: [Mousa.Khalafi@kashanu.ac.ir](mailto:Mousa.Khalafi@kashanu.ac.ir)

**Alexios Batrakoulis**, Department of Physical Education and Sport Science, University of Thessaly, Trikala, Greece; Email: [abatrakoulis@uth.gr](mailto:abatrakoulis@uth.gr)

**Supplementary table 1.** Risk of bias assessment

|  | Eligibility criteria specified | Subjects randomly allocated | Allocation method concealed | Groups similar at baseline | All subjects blinded | Therapists administering blinded | Blinding of Assessors | Outcomes obtained from >85% | Intention-to-Treat Analysis | Between-group results reported | Variability measures provided | Score | Score  (Qualitative) |
| --- | --- | --- | --- | --- | --- | --- | --- | --- | --- | --- | --- | --- | --- |
| Almenning et al. 2015 | ✓ | ✓ | ✓ | ✓ | 🗶 | 🗶 | ✓ | ✓ | 🗶 | ✓ | ✓ | 7 | Good |
| Benham et al. 2021 a | ✓ | ✓ | ✓ | 🗶 | 🗶 | 🗶 | ✓ | ✓ | ✓ | ✓ | ✓ | 7 | Good |
| Bruner et al. 2006 | ✓ | ✓ | ✓ | 🗶 | 🗶 | 🗶 | 🗶 | ✓ | 🗶 | ✓ | ✓ | 5 | Fair |
| Costa et al. 2018 | ✓ | ✓ | 🗶 | ✓ | 🗶 | 🗶 | 🗶 | ✓ | 🗶 | ✓ | ✓ | 5 | Fair |
| Elbandrawy et al. 2022 | ✓ | ✓ | 🗶 | ✓ | 🗶 | 🗶 | 🗶 | ✓ | 🗶 | ✓ | ✓ | 5 | Fair |
| Kiel et al. 2022 b | ✓ | ✓ | ✓ | ✓ | 🗶 | 🗶 | ✓ | ✓ | 🗶 | ✓ | ✓ | 7 | Good |
| Kirthika et al. 2019 | ✓ | ✓ | 🗶 | ✓ | 🗶 | 🗶 | 🗶 | ✓ | ✓ | ✓ | ✓ | 6 | Good |
| Konopka et al. 2015 | ✓ | ✓ | 🗶 | 🗶 | 🗶 | 🗶 | 🗶 | ✓ | 🗶 | ✓ | ✓ | 4 | Fair |
| Li et al. 2022 | ✓ | ✓ | ✓ | 🗶 | 🗶 | 🗶 | ✓ | 🗶 | 🗶 | ✓ | ✓ | 5 | Fair |
| Lionett et al, 2021 | ✓ | ✓ | 🗶 | 🗶 | 🗶 | 🗶 | 🗶 | ✓ | 🗶 | ✓ | ✓ | 4 | Fair |
| Lopes et al. 2018 | ✓ | ✓ | ✓ | 🗶 | 🗶 | 🗶 | 🗶 | 🗶 | ✓ | ✓ | ✓ | 5 | Fair |
| Mohammadi et al. 2023 | ✓ | ✓ | ✓ | ✓ | 🗶 | 🗶 | 🗶 | ✓ | 🗶 | ✓ | ✓ | 6 | Good |
| Nybacka et al. 2013 | ✓ | ✓ | ✓ | ✓ | 🗶 | 🗶 | 🗶 | 🗶 | 🗶 | ✓ | ✓ | 5 | Fair |
| Orio et al. 2016 | ✓ | ✓ | ✓ | ✓ | 🗶 | 🗶 | ✓ | ✓ | ✓ | ✓ | ✓ | 8 | Good |
| Patel et al. 2020 | ✓ | ✓ | ✓ | 🗶 | 🗶 | 🗶 | ✓ | 🗶 | 🗶 | ✓ | ✓ | 5 | Fair |
| Patten et al. 2022 | ✓ | ✓ | ✓ | 🗶 | 🗶 | 🗶 | 🗶 | 🗶 | ✓ | ✓ | ✓ | 5 | Fair |
| Philbois et al. 2022 | ✓ | ✓ | 🗶 | ✓ | 🗶 | 🗶 | 🗶 | ✓ | 🗶 | ✓ | ✓ | 5 | Fair |
| Ribeiro et al. 2020 | ✓ | ✓ | ✓ | 🗶 | 🗶 | 🗶 | 🗶 | 🗶 | 🗶 | ✓ | ✓ | 4 | Fair |
| Stener-Victorin et al.2009 | ✓ | ✓ | ✓ | 🗶 | 🗶 | 🗶 | ✓ | ✓ | 🗶 | ✓ | ✓ | 6 | Good |
| Thomson et al. 2008 | ✓ | ✓ | ✓ | 🗶 | 🗶 | 🗶 | 🗶 | 🗶 | 🗶 | ✓ | ✓ | 4 | Fair |
| Turan et al. 2015 | ✓ | ✓ | ✓ | 🗶 | 🗶 | 🗶 | 🗶 | ✓ | 🗶 | ✓ | ✓ | 5 | Fair |
| Vigorito et al. 2007 | ✓ | ✓ | 🗶 | ✓ | 🗶 | 🗶 | ✓ | ✓ | 🗶 | ✓ | ✓ | 6 | Good |
| Vizza et al. 2016 | ✓ | ✓ | ✓ | ✓ | 🗶 | 🗶 | 🗶 | ✓ | ✓ | ✓ | ✓ | 7 | Good |
| Wang et al. 2024 | ✓ | ✓ | ✓ | ✓ | 🗶 | 🗶 | 🗶 | 🗶 | 🗶 | ✓ | ✓ | 5 | Fair |
| Woodward et al. 2022 | ✓ | ✓ | ✓ | ✓ | 🗶 | 🗶 | 🗶 | ✓ | ✓ | ✓ | ✓ | 7 | Good |

(1) Eligibility Criteria specified, (2) Random allocation of participants, (3) Allocation concealed, (4) Groups similar at baseline, (5) Subjects blinded, (6) Therapists blinded, (7) Assessors blinded, (8) Outcome measures assessed in 85% of participants, (9) Intention to treat analysis, (10) Reporting of between group statistical comparison, (11) Point measures and measures of variability reported for main effects. low (✓), high (x) and unclear (?)

**Supplementary table 2**. Node-splitting method in comparison between direct and indirect evidence of different specific intervention for fasting glucose

| comparison | k | prop | nma | direct | indir | Diff | z | p-value |
| --- | --- | --- | --- | --- | --- | --- | --- | --- |
| AT:CON | 10 | 0.84 | -0.675 | 0.102 | -4.621 | 4.723 | 2.16 | 0.031 |
| AT:CT | 1 | 0.35 | -0.546 | -1.8 | 0.126 | -1.926 | -0.48 | 0.631 |
| AT:HIIT | 5 | 0.52 | -0.62 | -1.042 | -0.165 | -0.876 | -0.44 | 0.6591 |
| AT:Yoga/Tai Chi | 1 | 0.22 | 4.874 | 1.62 | 5.794 | -4.174 | -1.01 | 0.3112 |
| CT:CON | 3 | 0.9 | -0.129 | 0.14 | -2.638 | 2.778 | 0.45 | 0.6499 |
| HIIT:CON | 7 | 0.9 | -0.055 | -0.11 | 0.425 | -0.535 | -0.21 | 0.8347 |
| RT:CON | 2 | 0.92 | -0.053 | -1.098 | 11.418 | -12.516 | -1.46 | 0.1451 |
| Yoga/Tai Chi:CON | 1 | 0.82 | -5.549 | -6.3 | -2.126 | -4.174 | -1.01 | 0.3112 |
| HIIT:RT | 1 | 0.65 | -0.001 | -1.8 | 3.29 | -5.09 | -1 | 0.3154 |

Notes: p>0.05 indicates that indirect comparisons were consistent with direct comparisons.

**Supplementary table 3**. Node-splitting method in comparison between direct and indirect evidence of different specific intervention for fasting insulin

| comparison | k | prop | nma | direct | indir | Diff | z | p-value |
| --- | --- | --- | --- | --- | --- | --- | --- | --- |
| AT:CON | 10 | 0.88 | -0.259 | -0.197 | -0.699 | 0.502 | 1.05 | 0.2952 |
| AT:CT | 1 | 0.33 | 0.021 | 0.178 | -0.057 | 0.234 | 0.35 | 0.7249 |
| AT:HIIT | 5 | 0.65 | 0.123 | 0.012 | 0.33 | -0.318 | -0.77 | 0.4426 |
| CT:CON | 4 | 0.93 | -0.28 | -0.232 | -0.895 | 0.664 | 0.6 | 0.5465 |
| HIIT:CON | 7 | 0.81 | -0.382 | -0.4 | -0.307 | -0.093 | -0.2 | 0.8403 |
| RT:CON | 2 | 0.86 | -0.316 | -0.509 | 0.915 | -1.424 | -1.13 | 0.2568 |
| HIIT:RT | 1 | 0.47 | -0.066 | -0.405 | 0.236 | -0.64 | -0.72 | 0.4735 |

Notes: p>0.05 indicates that indirect comparisons were consistent with direct comparisons.

**Supplementary table 4.** Node-splitting method in comparison between direct and indirect evidence of different specific intervention for HOMA-IR

| comparison | k | prop | nma | direct | indir | Diff | z | p-value |
| --- | --- | --- | --- | --- | --- | --- | --- | --- |
| AT:CON | 8 | 0.88 | -0.575 | -0.463 | -1.389 | 0.927 | 1.14 | 0.2534 |
| AT:CT | 1 | 0.38 | -0.085 | 0.189 | -0.254 | 0.443 | 0.44 | 0.6595 |
| AT:HIIT | 3 | 0.57 | -0.011 | -0.162 | 0.188 | -0.351 | -0.51 | 0.6105 |
| CT:CON | 3 | 0.92 | -0.49 | -0.381 | -1.673 | 1.293 | 0.81 | 0.4172 |
| HIIT:CON | 6 | 0.85 | -0.564 | -0.56 | -0.583 | 0.023 | 0.03 | 0.9784 |
| RT:CON | 1 | 0.77 | -0.506 | -0.894 | 0.822 | -1.716 | -0.94 | 0.3465 |
| HIIT:RT | 1 | 0.77 | -0.057 | -0.447 | 1.265 | -1.712 | -0.94 | 0.3465 |

Notes: p>0.05 indicates that indirect comparisons were consistent with direct comparisons.

**Supplementary table 5.** Node-splitting method in comparison between direct and indirect evidence of different specific intervention for TG

| comparison | k | prop | nma | direct | indir. | Diff | z | p-value |
| --- | --- | --- | --- | --- | --- | --- | --- | --- |
| AT:CON | 6 | 0.92 | 0.5 | 0.176 | 4.485 | -4.308 | -0.29 | 0.7682 |
| AT:CT | 1 | 0.02 | 4.113 | 15.06 | 3.836 | 11.224 | 0.43 | 0.6677 |
| AT:HIIT | 3 | 0.17 | 1.845 | 7.543 | 0.673 | 6.87 | 0.62 | 0.5326 |
| CT:CON | 2 | 1 | -3.613 | -3.593 | -31.582 | 27.989 | 0.58 | 0.5612 |
| HIIT:CON | 5 | 0.99 | -1.345 | -1.194 | -14.672 | 13.479 | 0.83 | 0.4069 |
| RT:CON | 1 | 1 | -4.106 | -4.42 | 71.715 | -76.135 | -0.35 | 0.7254 |
| HIIT:RT | 1 | 0.54 | 2.761 | -1.78 | 8.079 | -9.859 | -0.35 | 0.7254 |

Notes: p>0.05 indicates that indirect comparisons were consistent with direct comparisons.

**Supplementary table 6.** Node-splitting method in comparison between direct and indirect evidence of different specific intervention for TC

| comparison | k | prop | nma | direct | indir. | Diff | z | p-value |
| --- | --- | --- | --- | --- | --- | --- | --- | --- |
| AT:CON | 8 | 0.89 | -2.69 | -2.81 | -1.696 | -1.114 | -0.09 | 0.9253 |
| AT:CT | 1 | 0.27 | 1.865 | 2.32 | 1.7 | 0.62 | 0.04 | 0.9653 |
| AT:HIIT | 3 | 0.52 | 0.772 | 3.322 | -2.011 | 5.333 | 0.58 | 0.5616 |
| CT:CON | 2 | 0.95 | -4.555 | -4.861 | 0.912 | -5.773 | -0.24 | 0.8124 |
| HIIT:CON | 5 | 0.91 | -3.463 | -2.427 | -14.356 | 11.928 | 0.91 | 0.3613 |
| RT:CON | 1 | 0.76 | -2.089 | 3.87 | -20.545 | 24.415 | 1.05 | 0.2937 |
| HIIT:RT | 1 | 0.82 | -1.373 | 3.49 | -23.678 | 27.168 | 1.05 | 0.2937 |

Notes: p>0.05 indicates that indirect comparisons were consistent with direct comparisons.

**Supplementary table 7.** Node-splitting method in comparison between direct and indirect evidence of different specific intervention for LDL

| comparison | k | prop | nma | direct | indir. | Diff | z | p-value |
| --- | --- | --- | --- | --- | --- | --- | --- | --- |
| AT:CON | 8 | 0.93 | -1.143 | -1.116 | -1.484 | 0.368 | 0.04 | 0.9701 |
| AT:CT | 1 | 0.22 | 7.262 | -0.39 | 9.434 | -9.824 | -0.98 | 0.3267 |
| AT:HIIT | 3 | 0.44 | 5.316 | 7.297 | 3.789 | 3.507 | 0.52 | 0.6007 |
| HIIT:CON | 5 | 0.93 | -6.459 | -5.947 | -13.216 | 7.269 | 0.71 | 0.4756 |
| RT:CON | 1 | 0.89 | -18.708 | -15.48 | -44.286 | 28.806 | 0.65 | 0.5167 |
| HIIT:RT | 1 | 0.89 | 12.248 | 15.48 | -13.3 | 28.78 | 0.65 | 0.5167 |

Notes: p>0.05 indicates that indirect comparisons were consistent with direct comparisons.

**Supplementary table 8.** Node-splitting method in comparison between direct and indirect evidence of different specific intervention for HDL

| comparison | k | prop | nma | direct | indir. | Diff | z | p-value |
| --- | --- | --- | --- | --- | --- | --- | --- | --- |
| AT:CON | 8 | 0.88 | 3.036 | 3.366 | 0.632 | 2.734 | 0.55 | 0.5835 |
| AT:CT | 1 | 0.42 | 2.047 | 0 | 3.544 | -3.544 | -0.67 | 0.5025 |
| AT:HIIT | 3 | 0.58 | 0.845 | -0.853 | 3.148 | -4.001 | -0.96 | 0.3368 |
| CT:CON | 2 | 0.91 | 0.989 | 0.377 | 7.145 | -6.768 | -0.84 | 0.3999 |
| HIIT:CON | 5 | 0.89 | 2.191 | 1.715 | 5.882 | -4.166 | -0.75 | 0.4503 |
| RT:CON | 1 | 0.85 | -2.732 | -1.43 | -10.209 | 8.779 | 0.67 | 0.5017 |
| HIIT:RT | 1 | 0.58 | 4.922 | 7.63 | 1.148 | 6.482 | 0.67 | 0.5017 |

Notes: p>0.05 indicates that indirect comparisons were consistent with direct comparisons.

**Supplementary table 9.** Node-splitting method in comparison between direct and indirect evidence of different specific intervention for CRP

| comparison | k | prop | nma | direct | indir. | Diff | z | p-value |
| --- | --- | --- | --- | --- | --- | --- | --- | --- |
| HIIT:CON | 2 | 0.95 | -0.292 | -0.264 | -0.816 | 0.552 | 0.31 | 0.757 |
| RT:CON | 2 | 0.93 | 0.007 | -0.048 | 0.712 | -0.76 | -0.47 | 0.6387 |
| HIIT:RT | 1 | 0.63 | -0.299 | -0.486 | 0.023 | -0.509 | -0.5 | 0.6189 |

Notes: p>0.05 indicates that indirect comparisons were consistent with direct comparisons.

**Supplementary Figure 1.** Forest plot of the effects of exercise training on fasting glucose. Data are reported as WMD (95% confidence limits). WMD: weighted mean difference.

**Supplementary Figure 2.** Forest plot of the effects of exercise training on fasting insulin. Data are reported as SMD (95% confidence limits). WMD: standardized mean difference.

**Supplementary Figure 3.** Forest plot of the effects of exercise training on insulin resistance. Data are reported as SMD (95% confidence limits). SMD: standardized mean difference.

**Supplementary Figure 4.** Forest plot of the effects of exercise training on TG. Data are reported as WMD (95% confidence limits). WMD: weighted mean difference.

**Supplementary Figure 5.** Forest plot of the effects of exercise training on TC. Data are reported as WMD (95% confidence limits). WMD: weighted mean difference.

**Supplementary Figure 6.** Forest plot of the effects of exercise training on LDL. Data are reported as WMD (95% confidence limits). WMD: weighted mean difference.

**Supplementary Figure 7.** Forest plot of the effects of exercise training on HDL. Data are reported as WMD (95% confidence limits). WMD: weighted mean difference.

**Supplementary Figure 8.** Forest plot of the effects of exercise training on CRP. Data are reported as SMD (95% confidence limits). SMD: standardized mean difference.


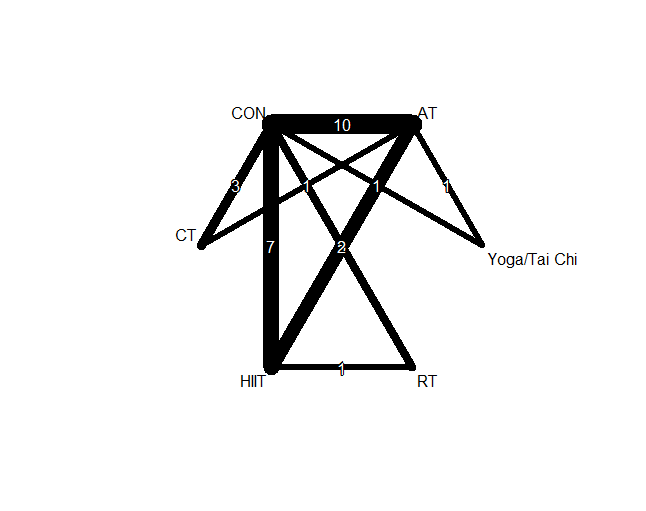
 **Supplementary Figure 9.** Network geometric map of studies investigating the effect of exercise training on fasting glucose


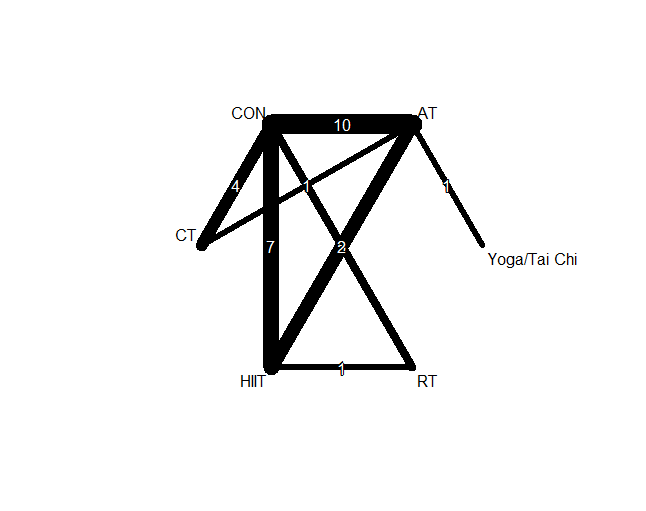
 **Supplementary Figure 10.** Network geometric map of studies investigating the effect of exercise training on fasting insulin


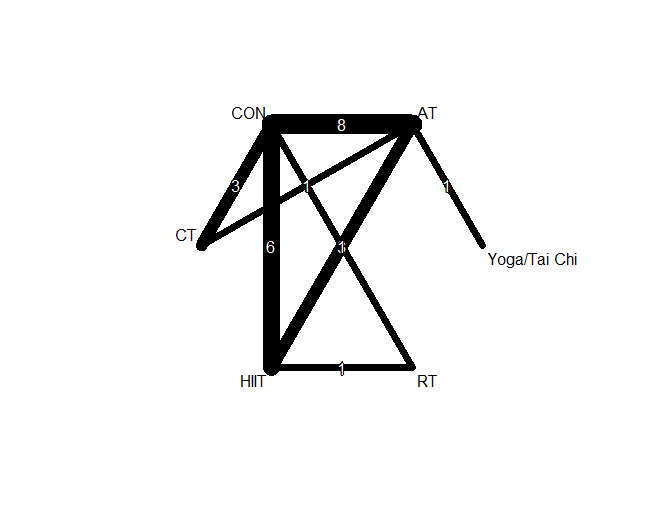
 **Supplementary Figure 11.** Network geometric map of studies investigating the effect of exercise training on insulin resistance


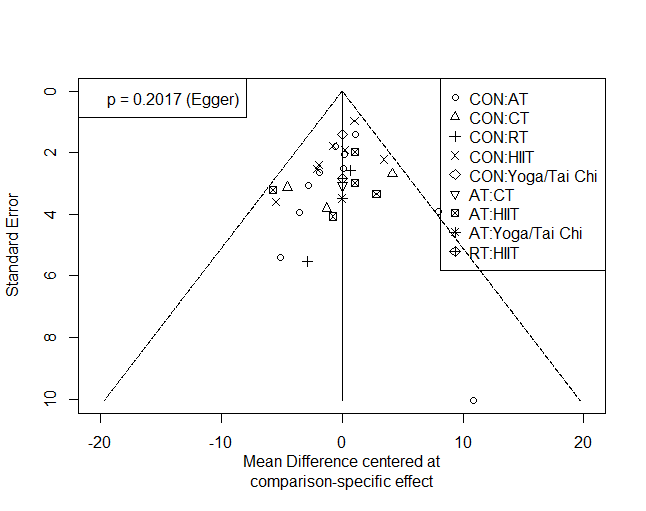


**Supplementary Figure 12.** Network meta-analysis of funnel plots for fasting glucose


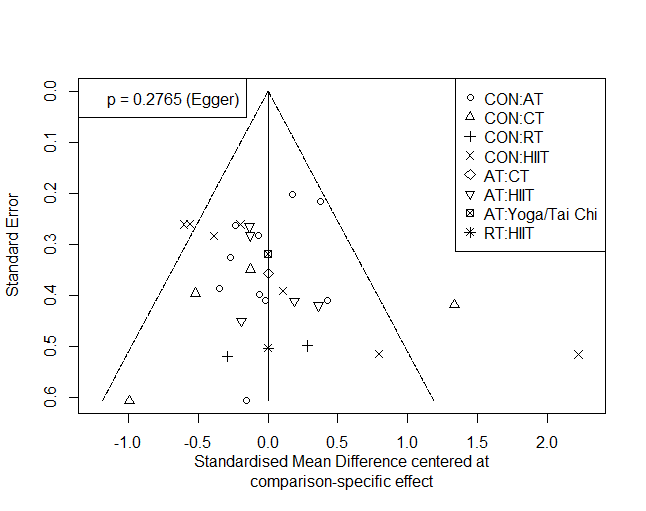
 **Supplementary Figure 13.** Network meta-analysis of funnel plots for fasting insulin


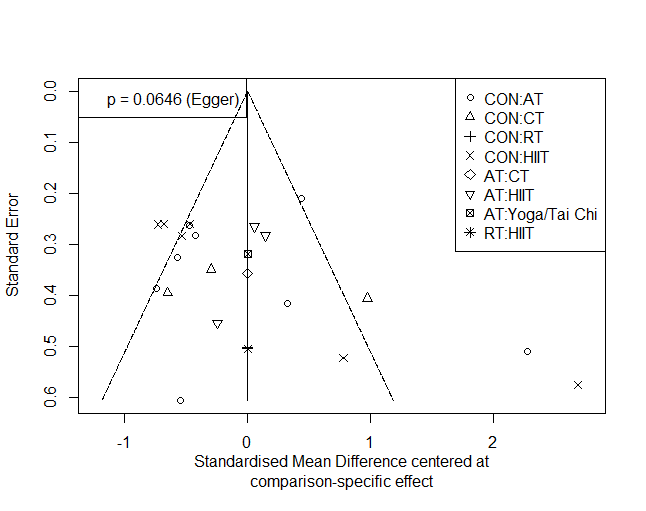
 **Supplementary Figure 14.** Network meta-analysis of funnel plots for insulin resistance


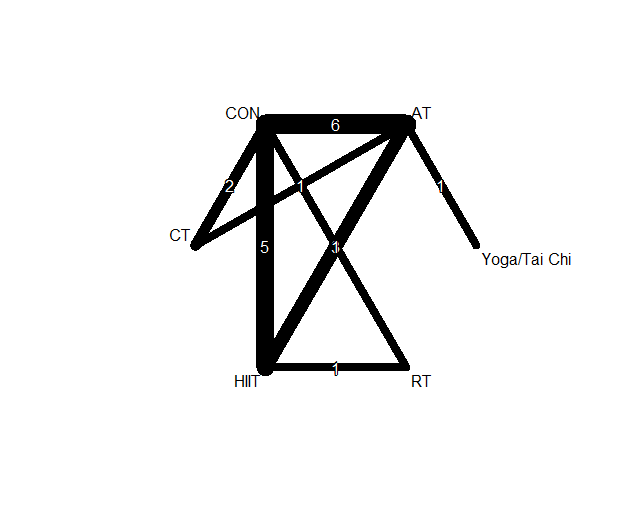
 **Supplementary Figure 15.** Network geometric map of studies investigating the effect of exercise training on TG


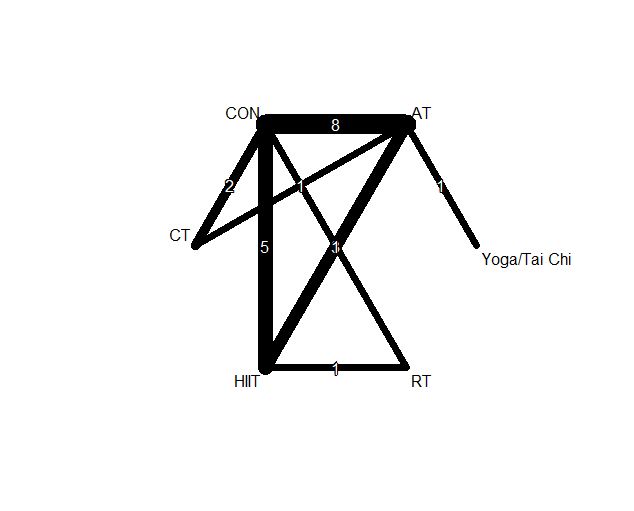
 **Supplementary Figure 16.** Network geometric map of studies investigating the effect of exercise training on TC


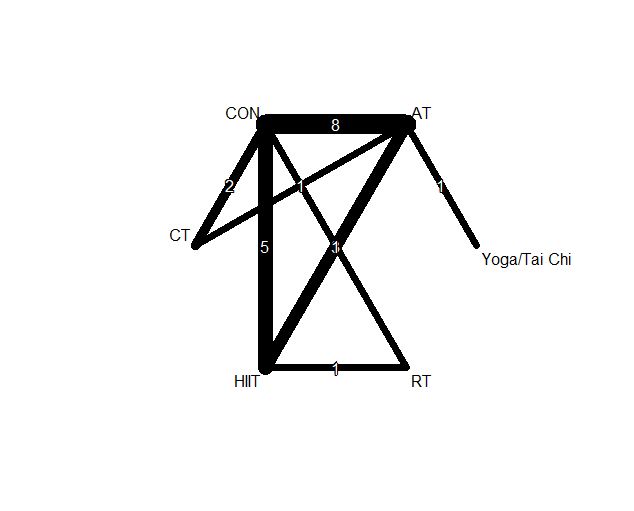
 **Supplementary Figure 17.** Network geometric map of studies investigating the effect of exercise training on LDL


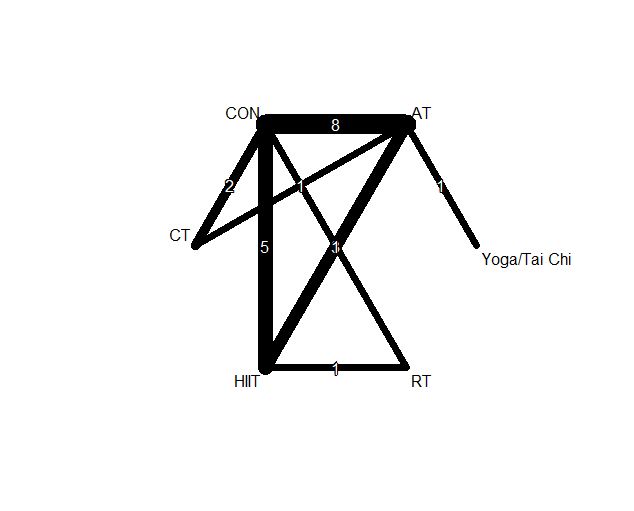
 **Supplementary Figure 18.** Network geometric map of studies investigating the effect of exercise training on HDL


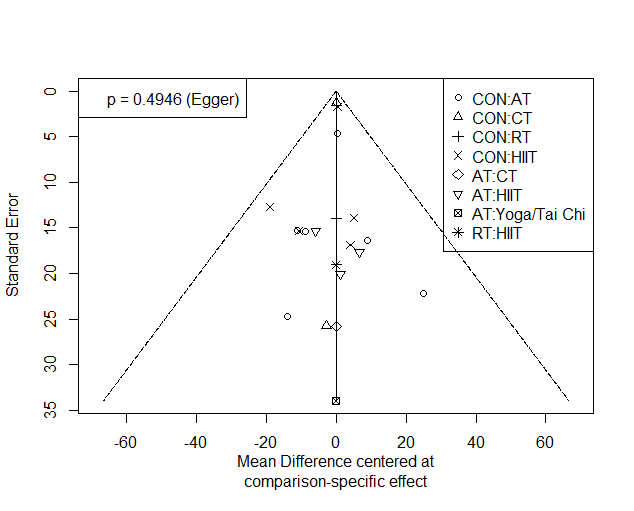
 **Supplementary Figure 19.** Network meta-analysis of funnel plots for TG


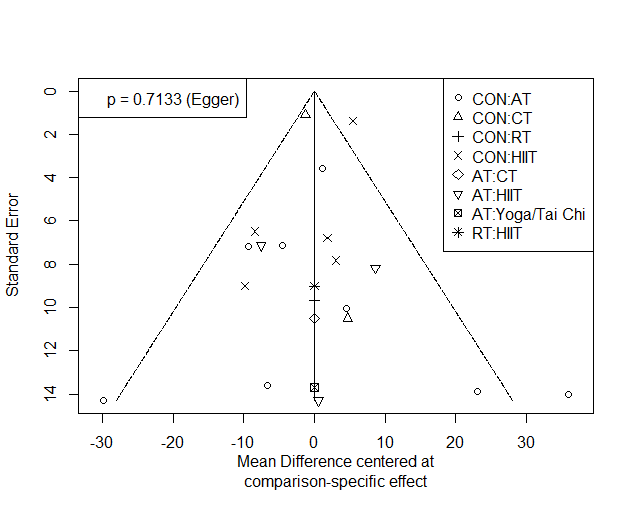
 **Supplementary Figure 20.** Network meta-analysis of funnel plots for TC


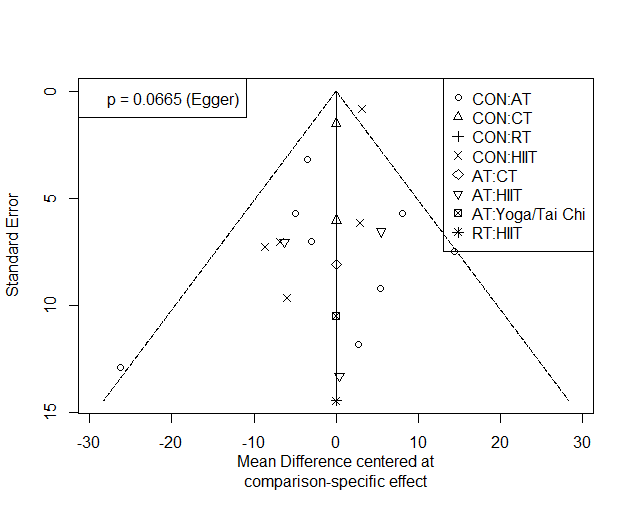
 **Supplementary Figure 21.** Network meta-analysis of funnel plots for LDL


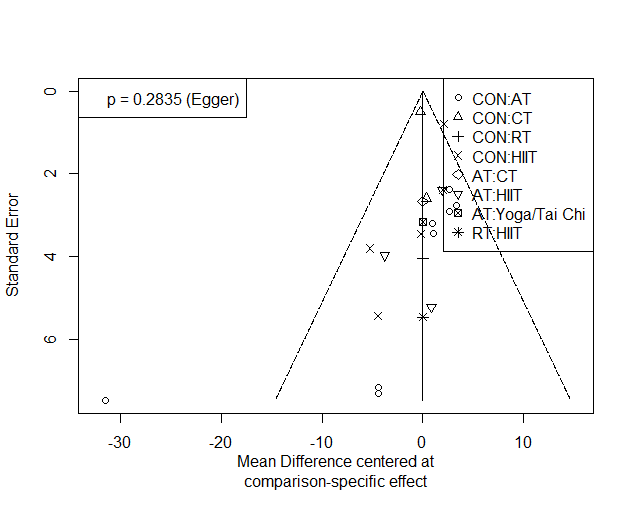
 **Supplementary Figure 22.** Network meta-analysis of funnel plots for HDL


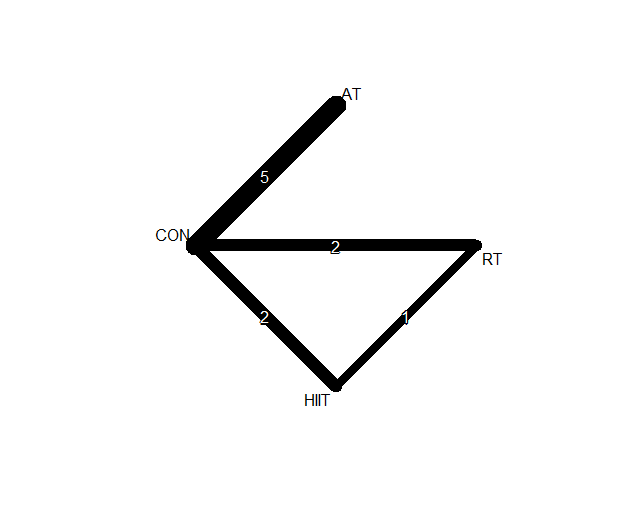
 **Supplementary Figure 23.** Network geometric map of studies investigating the effect of exercise training on CRP


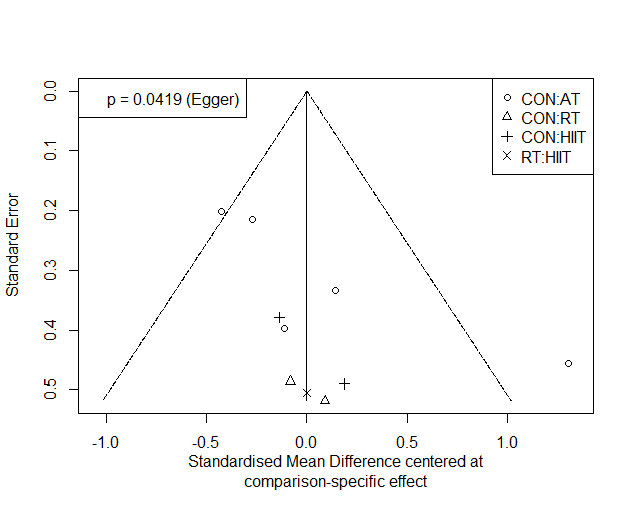
 **Supplementary Figure 24.** Network meta-analysis of funnel plots for CRP
